# Generation and validation of ActiGraph GT3X+ accelerometer cut-points for assessing physical activity intensity in older adults. The OUTDOOR ACTIVE validation study

**DOI:** 10.1371/journal.pone.0252615

**Published:** 2021-06-03

**Authors:** Karin Bammann, Nicola K. Thomson, Birte Marie Albrecht, Duncan S. Buchan, Chris Easton

**Affiliations:** 1 Working group Epidemiology of Demographic Change, Institute for Public Health and Nursing Sciences (IPP), University of Bremen, Bremen, Germany; 2 Institute for Clinical Exercise and Health Sciences, University of the West of Scotland, Lanarkshire, United Kingdom; Linneaus University, SWEDEN

## Abstract

The study of physical activity in older adults is becoming more and more relevant. For evaluation of physical activity recommendations, intensity-specific accelerometer cut-points are utilized. However, research on accelerometer cut-points for older adults is still scarce. The aim of the study was to generate placement-specific cut-points of ActiGraph GT3X+ activity counts and raw measures of acceleration to determine physical activity intensity in older adults. A further aim was to compare the validity of the generated cut-points for a range of different physical activities. The study was a single experimental trial using a convenience sample. Study participants were 20 adults aged 59 to 73 years. Accelerometers were worn at six different placements (one on each wrist, one on each ankle, and two at the hip) and breath-by-breath indirect calorimetry was used as the reference for energy. The experiment comprised of two parts; a) The first required participants to walk on a treadmill at incremental speeds (3.0–5.0 km·h^-1^), and b) Five different everyday activities (reading, cleaning, shopping, cycling, aerobics) were staged in the laboratory setting. Accelerometer cut-points (activity counts, raw data) were derived for each of the investigated placements by linear regression using the treadmill part. Performance of the cut-points was assessed by applying the cut-points to the everyday activities. We provide cut-points for six placements and two accelerometer metrics in the specific age group. However, the derived cut-points did not outperform published ones. More research and innovative approaches are needed for improving internal and external validity of research results across populations and age groups.

## Introduction

In humans, physical activity (PA) is essential for optimal functioning of musculoskeletal and digestive systems and also for circulation [1]. In this context, PA of sufficient volume and intensity can increase life expectancy, motor skills, overall well-being and quality of life [2]. In 2009, three million premature deaths world-wide were attributed to a lack of sufficient PA [1]. In the European Union, the main causes of death are diseases of the cardiovascular system for which physical inactivity is one of the major risk factors [2, 3]. Therefore, the World Health Organization recommends 150 minutes of moderate or 75 minutes of vigorous PA per week in older adults [4]. By this definition, 35% of European adults are considered as physically inactive and this proportion increases with age [1]. A previous study demonstrated that both moderate and high intensity exercise can lead to considerable improvements in physiological fitness and health related quality of life in previously sedentary older adults [5].

The intensity of PA is defined by the metabolic equivalent (MET), which is determined by relating energy expenditure (EE) of the activity to body mass [6]. Thus, for assessing PA recommendations in epidemiological studies, measurement of EE is essential. The gold standard methods for measuring EE are direct calorimetry and the doubly labelled water method, however, these are expensive and place a high burden on the study participants which limits their use in population studies [7, 8]. Alternatively, accelerometry has been suggested as a cheaper and more practical method for estimating EE in larger epidemiological studies [9, 10]. The tri-axial ActiGraph GT3X+ accelerometer (ActiGraph, FL, USA) records accelerations and decelerations of the body in three different planes of motion of up to 100 times every second. Laboratory based studies have found positive relationships between accelerometer output and activity EE [11].

Several studies have published accelerometer cut-points to classify PA intensity levels in adults. These are often derived from treadmill activities in a laboratory setting [12–14] and, therefore, lack ecological validity. The cut-points might not hold for unstaged acitivities as these are often associated with higher metabolic costs than in a controlled laboratory setting [15]. Moreover, most studies with an adult population do not specifically address older adults. Changing gait patterns with age [16] and a different range of typical activities limit the transferability of these cut-points for older adults [17, 18]. In addition, cut-points are specific to the type of accelerometer and placement at the body, and research on wrist- or ankle-worn accelerometry in older adults is scarce.

Studies suggest that the accuracy of EE estimation depends on accelerometer placement and this association varies for different activities and activity speeds [19]. For example, ankle placement might be more suitable for walking, running, or cycling [20], while wrist placement has been shown to better measure upper body everyday activities such as washing dishes [21]. Wrist placement also increases compliance in comparison to hip placement [22], and thus, can help reduce selection bias in population-based studies. Furthermore, accelerometers can be worn on the wrist during sleep with minimal discomfort unlike hip-worn accelerometers, which can facilitate full-day measurements and reduce the extent of missing data [23].

The aim of the study was to generate placement-specific cut-points of ActiGraph GT3X+ activity counts and raw measures of acceleration to determine PA intensities in older adults. A further aim was to compare the validity of the generated cut-points for a range of different activities simulated in a control laboratory setting.

## Methods

### Ethical approval

Ethical approval was obtained from the School of Science and Sport Ethics Committee of the University of the West of Scotland. Written informed consent was obtained from each participant and participants were informed of their right to withdraw at any time without explanation.

### Study population and design

The study is a sub-component of the OUTDOOR ACTIVE study which focuses on community-based participatory PA interventions for older adults in an urban setting in Germany [24, 25]. The cut-points were generated for the future evaluation of the PA interventions in the OUTDOOR ACTIVE cluster-randomized trial [25].

The study population consisted of a convenience sample of twenty healthy older adults, eleven males and nine females (mean ± SD: 62.9 ± 3.6 years old). The laboratory work took place at the University of the West of Scotland, Lanarkshire campus, Scotland from 06/2016-12/2016. Participants were recruited using a snowballing system. Initially, the study was advertised by word-of-mouth and on social media sites (Facebook and Twitter). Moreover, participants were encouraged to bring relatives or peers as new participants. Included were non-institutionalized adults aged 55 to 75 years of age. Exclusion criteria were a history of cardiorespiratory disease, neurological disease or elevated blood pressure. The de-anonymized study data set can be found in S1 Appendix.

### Experimental design

Upon arrival, participants were briefed on the study protocol (see S2 Appendix). The experiment was conducted in two parts. In the *treadmill part*, participants walked continuously for 20 minutes on a treadmill set at 1% gradient, to mimic the energetic and metabolic cost of outdoor walking [26]. The initial speed was set at 3.0 km·h^-1^. The speed was increased incrementally by 0.5 km·h^-1^ every 4 minutes to a maximum speed of 5.0 km·h^-1^, or until the participant was not able to maintain the walking speed.

The *everyday activities part* consisted of five staged activities performed consecutively in a pre-set order for a maximum period of 4 minutes with at least 2 minutes rest between each activity. The participants received gentle verbal encouragement throughout each activity. The activities were:

- Reading: This activity was staged by lying on a bed reading a newspaper/magazine. The reading activity was measured prior to the treadmill part of the study.
- Shopping: This activity was staged by carrying a plastic bag with a self-selected weight, comparable to what they would carry in a bag of groceries when shopping at home (between 2–4 kg), while walking on the treadmill. The treadmill was set to an incline of 1% with an initial speed of 2.5 km·h^-1^. Participants were then asked to self-select a comfortable speed that would emulate their habitual walking speed.
- Cleaning: This activity was staged by brushing with a broom on a marked area at the laboratory at self-paced intensity.
- Cycling: This activity was staged by cycling on an ergometer at self-paced intensity.
- Aerobic: This activity was staged by simulating an aerobics class for older adults following a member of the research team. Within the four minutes, eight different easy, low-impact exercises were performed.

### Measurements

Prior to the trial, body height was measured using a Seca 213 portable stadiometer (Seca GmbH & Co. KG, Hamburg, Germany) and body mass was measured using a Seca clara 803 digital scale (Seca GmbH & Co. KG, Hamburg, Germany). Blood pressure was measured using an Omron M6 digital blood pressure monitor (Omron Healthcare, Inc., Kyoto, Japan). A Polar H7 monitor (Polar Electro Oy, Kempele Finland) was attached to the participant’s chest in order to continuously monitor HR.

Breath-by-breath pulmonary gas exchange was measured continuously throughout all activities using indirect calorimetry (Ultima CPX, MedGraphics, MN, USA). Prior to the arrival of each participant, the Ultima CPX was calibrated using a 3L syringe (volume) and reference gases (oxygen and carbon dioxide) according to the manufacturer’s guidelines.

Before each trial, tri-axial GT3X+ accelerometers (ActiGraph, FL, USA) were initialized to record data at a sampling frequency of 30Hz in three axes: vertical, mediolateral and anteroposterior, using ActiLife software (V6.13.3 Lite Edition, ActiGraph, FL, USA). The accelerometers were synchronized to the clock of the computer used in the experiments. The accelerometers were worn on the dorsal surface in between the ulnar and radial styloid processes of both wrists, the anterior-superior iliac spine of the right hip (correct placement), and superior to the lateral malleolus of both ankles. To check whether exact hip-placement is vital for the accelerometer’s validity, the last device was placed anywhere above the hip (erroneous placement). The hip and wrist accelerometers were attached to an elastic, nylon strap and the ankle devices were secured in place with an elastic bandage. The accelerometers were worn throughout the entire trial. Data was downloaded and aggregated into 1-minute epochs.

### Data processing and statistical analyses

The ActiGraph.gt3x files containing count values and raw accelerometer signals for each of the three axes were converted to time-stamp free.csv files which were exported to R v3.5.3 (R Foundation for Statistical Computing, Vienna, Austria, https://cran.r-project.org/).

Vector magnitudes (VM) were calculated using the count values of the three axes X, Y, and Z by

$$VM=\sqrt{X^{2}+Y^{2}+Z^{2}}.$$

To derive *raw accelerations*, the.csv files were processed using the GGIR package V1.9.1 which auto-calibrated the raw tri-axial accelerometer signals and computed average acceleration expressed as Euclidean Norm Minus One (ENMO) which were subsequently converted to milli-gravitational units (m*g*) per 1-s epochs, with negative values rounded up to zero and corrected for gravity [27]. The package regenerated the time-stamps with files subsequently exported to Microsoft Excel 2016 (Microsoft, Redmond, WA). When there was a lack of sufficient non-movement periods available for auto-calibrating the accelerometer data in GGIR, we used back-up calibration coefficients derived from free-living data collected with the same accelerometer unit as reported elsewhere [28, 29]. Finally, no accelerometer files were excluded from subsequent analyses since post-calibration error was < 0.01 g.

For each participant, the data (activity counts and ENMO) was time-matched with the protocol. For each activity, the following procedure was performed: For each axis, the last minute of the activity (60 measurement points) were used for calculation of average values per second and multiplied by 60 to obtain values per minute.

METs were used to express intensity of activities as multiples of the resting or 1 MET value. MET values were calculated by dividing the mean rate of oxygen utilization ($\dot{V}O_{2}$ ml·kg^-1^·min^-1^) from the last two minutes of each stage, by 3.5 ml·kg^-1^·min^-1^, commonly used to define 1 MET [30]. MET intensity was classified as < 3 METs for sedentary/light, 3 - < 6 METS for moderate and ≥ 6 METs for vigorous intensity [31].

Body mass index (BMI) was calculated dividing body weight in kg by squared body height in m^2^. Means and standard deviations were computed for description of the data.

PA intensity accelerometer cut-points for moderate and vigorous PA were derived by linear regression with $\dot{V}O_{2}$ as an independent variable and VM counts [ENMO] as dependent variables using all single observations of the treadmill part of the study (five treadmill activities per 20 participants; n = 95, for missing values see S3 Appendix). Cut-points were determined as predicted values at 3 METs (for moderate intensity) resp. 6 METs (for vigorous intensity). For each of the models, confounding of age and sex was tested at α = 0.05 in a separate linear regression model with the residual as dependent variable. Additionally, the explained variance was reported using the coefficient of determination R^2^.

To compare performance of accelerometer placements for everyday activities, the correlation of VM counts [ENMO] with $\dot{V}O_{2}$ during the activity was calculated using Pearson’s correlation coefficients (five staged activities per 20 participants; n = 99, for missing value see S2 Appendix). To assess the performance of the derived accelerometer cut-points, intraclass correlation coefficients were calculated using absolute agreement of two-way mixed single measures (ICC (3,1), absolute) [32]. The basis for calculating the ICCs were the PA intensities as predicted by the newly-derived cut-points on the one hand, and the PA intensity levels as measured during activity (empirical level) on the other hand. It can take values between 0 (no agreement) and 1 (absolute agreement).

All statistical analyses were performed using the statistical analysis software IBM SPSS statistics for Windows, version 20.0 (IBM, New York (NY), USA). Data pre-processing was undertaken using ActiLife V6.13.3 (ActiGraph, FL, USA), R (R Foundation for Statistical Computing, Vienna, Austria) and Microsoft Excel (Microsoft, Redmond (WA), USA).

## Results

### Description of the study sample

The age of the 20 study participants ranged from 59 to 73 years, with 45% women (see Table 1). The BMI ranged from 21.2 to 43.0 kg/m^2^ with women having a higher BMI than men (27.7 kg/m^2^ vs. 25.9 kg/m^2^). $\dot{V}O_{2}$ during the reading activity ranged from 2.2 ml·kg^-1^·min^-1^ to 3.9 ml·kg^-1^·min^-1^ in the participants. Average METs in the staged free-living activities in both sexes ranged from 0.9 METs in reading newspaper to 4.7 METs in cycling on an ergometer, with higher METs in men than in women in most of the activities.

**Table 1:** Description of the study sample (means and standard deviations).

	Men (n = 11)	Women (n = 9)	Total (n = 20)
Age (years)	63.9 (4.3)	61.7 (2.1)	62.9 (3.6)
Body height (cm)	173.9 (4.2)	161.7 (7.4)	168.4 (8.4)
Body weight (kg)	78.6 (14.3)	72.0 (16.0)	75.6 (15.1)
Body mass index (kg/m^2^)	25.9 (4.3)	27.7 (6.6)	26.7 (5.4)
Resting heart rate (bpm)	61.0 (8.5)	61.7 (8.9)	61.3 (8.4)
Systolic blood pressure (mmHg)	135.6 (11.2)	133.9 (17.2)	134.9 (13.9)
Diastolic blood pressure (mmHg)	81.6 (7.2)	80.0 (12.5)	80.9 (9.7)
Oxygen utilization while reading (ml·kg^-1^·min^-1^)	3.4 (0.6)	3.1 (0.4)	3.3 (0.5)
METs in kcal·kg^−1^·h^−1^ during staged activities			
*Reading*	1.0 (0.2)	0.9 (0.1)	0.9 (0.1)
*Cleaning*	3.2 (1.2)	2.9 (0.2)	3.0 (0.9)
*Shopping*	3.4 (0.7)	3.4 (0.2)	3.4 (0.2)
*Aerobics*	4.8 (1.2)	4.3 (0.4)	4.6 (1.0)
*Cycling*	5.0 (2.3)	4.4 (0.5)	4.7 (1.7)

MET metabolic equivalent of task

### Derivation of cut-points of treadmill data

Cut-points derived from the treadmill tests varied considerably by placement of the accelerometer (see Table 2 for VM counts, Table 3 for ENMO; and S4 Appendix for mean values and standard deviations of the measurements). For ankle placement, sex-specific cut-points were derived since sex (p<0.001) and age (p<0.01) were statistically significantly associated with the residuals of the regression model estimated in the total group of participants. For all other placements, sex and age were not associated to the residuals. The proportion of explained variance was highest for erroneous hip (45% for VM counts resp. 46% for ENMO) and hip (37% resp. 33%) placement, and ankle placement in women only (51% resp. 48% dominant ankle and 60% resp. 54% non-dominant ankle). Contrastingly, the explained variance for ankle placement was very low in men.

**Table 2:** Accelerometer cut-points for moderate and vigorous physical activity derived from treadmill experiments. Vector magnitudes of 60-sec intervals sampled at 1-sec epochs.

Placement	Sex	VM cut-point moderate PA intensity (3-<6 METs)	VM cut-point vigorous PA intensity (≥6 METs)	R^2^
**Ankle dominant**	**Male**	8829	12482	0.08
	**Female**	9365	20962	0.51
**Ankle non-dominant**	**Male**	8636	13242	0.14
	**Female**	9460	21683	0.60
**Wrist dominant**	**All**	3997	8391	0.12
**Wrist non-dominant**	**All**	3268	7890	0.17
**Hip correct**	**All**	2857	6799	0.37
**Hip erroneous**	**All**	2811	7567	0.45

* All prediction equations and cut-points are based on the vector magnitude

MET metabolic equivalent of task

PA physical activity

VM vector magnitude

**Table 3:** Accelerometer cut-points for moderate and vigorous physical activity derived from treadmill experiments. Euclidian Norm Minus One in milligravity of 60-sec intervals sampled at 1-sec epochs.

Placement	Sex	ENMO cut-point moderate PA intensity (3-<6 METs)	ENMO cut-point vigorous PA intensity (≥6 METs)	R^2^
** Ankle dominant**	**Male**	323	520	0.11
	**Female**	361	866	0.48
**Ankle non-dominant**	**Male**	316	484	0.08
	**Female**	346	880	0.54
**Wrist dominant**	**All**	122	234	0.07
**Wrist non-dominant**	**All**	100	245	0.14
**Hip correct**	**All**	82	191	0.33
**Hip erroneous**	**All**	94	230	0.46

* All prediction equations and cut-points are based on ENMO values

ENMO Euclidian norm minus one

MET metabolic equivalent of task

PA physical activity

### Test of derived cut-points by staged activity data

Performance of the different accelerometer placements was dependent on activity type. For cleaning and aerobics, accelerometer measurements correlated highest with $\dot{V}O_{2}$ in wrist placements (r = 0.74 (VM counts); r = 0.65 (ENMO)). Ankle (r = 0.44 (VM counts), r = 0.35 (ENMO)) and hip (r = 0.30 (VM counts), r = 0.40 (ENMO)) placement had the highest correlations during the shopping activity and ankle placement performed best during cycling (r = 0.83 (VM counts), r = 0.86 (ENMO)). $\dot{V}O_{2}$ during reading did not correlate with accelerometer output in any of the investigated placements. Of note, accelerometer output correlated highly between dominant and non-dominant limbs (wrist: r = 0.95 (VM counts), r = 0.93 (ENMO); ankle: r > 0.99 (VM counts), r = 0.99 (ENMO) and between correct and erroneous hip placement (r = 0.90 (VM counts), r = 0.86 (ENMO)) (see S5 Appendix).

The cut-points derived from the treadmill tests were tested using the staged everyday activities (Table 4). Using VM counts, intraclass correlation of PA intensity classification by accelerometer was moderate for ankle placement (ICC = 0.59), and poor for all other placements. For ENMO cut-points, intraclass correlation was poor regardless of placement. Whether the activity intensity is correctly classified depends on the type of activity with misclassification being worst for cycling. The intensity of the cycling activity that was performed (moderate intensity by 17 of the 20 participants) was severely underestimated using hip and wrist placement, which placed most of the participants in the sedentary/light intensity category. For ankle placement, VM counts overestimated and ENMO counts underestimated the intensity. A similar pattern was observed in the cleaning category (underestimation for ankle and hip placement, overestimation for wrist placement). The intensity of the shopping activity, which involved weight carrying, was generally underestimated by accelerometer irrespective of the placement. When omitting the cycling activity, intraclass correlation improved for hip and wrist placement. In this case, a moderate correlation was found for ankle and wrist using VM counts, and for wrist using ENMO. The comparison of VM counts and ENMO revealed that intraclass correlation was highest for wrist placement (r = 0.88) followed by hip placement (r = 0.66), and lowest for ankle placement (r = 0.34).

**Table 4:** Robustness of derived cut-points tested in all staged free-living activities all placements.

	Reference	Accelerometer placement					
		Ankle^§^		Hip^#^		Wrist^§^	
	VO_2_	VM	ENMO	VM	ENMO	VM	ENMO
	Proportions of physical activity intensities (sedentary/light-moderate-vigorous)						
Reading	100-0-0	100-0-0	100-0-0	100-0-0	100-0-0	90-10-0	100-0-0
Cleaning	55-45-0	100-0-0	100-0-0	100-0-0	100-0-0	0-50-50	20-60-20
Shopping	22-78-0	53-42-5	37-58-5	47-53-0	58-42-0	47-53-0	47-47-5
Cycling	5-85-10	0-45-55	65-25-10	90-10-0	95-5-0	100-0-0	100-0-0
Aerobics	0-85-15	10-70-20	90-10-0	35-55-10	65-35-0	0-15-85	10-15-75
**Overall**	**37-58-5**	**53-31-16**	**79-18-3**	**73-25-2**	**84-16-0**	**48-25-27**	**56-24-20**
	Intraclass correlation (2-way-mixed) for classification by ..						
.. accelerometer and reference	-	0.59	0.24	0.25	0.17	0.30	0.40
.. accelerometer and reference (w/o Cycling)	-	0.58	0.13	0.41	0.30	0.50	0.61
.. accelerometer VM and accelerometer ENMO	-	0.34		0.66		0.88	

§ Placement at non-dominant limb

# correct placement

ENMO Euclidian norm minus one

VM Vector magnitude

## Discussion

The current study provides accelerometer cut-points for VM counts and ENMO for different accelerometer placements in older adults. The cut-points were cross-validated for five free-living activities. In the following, cut-points, metrics, and placements are discussed in more detail.

### Accelerometer cut-points and metrics

Prior to this study, VM count cut-points for the ActiGraph GT3X+ were available for the hip in older adults [12, 33] but not for other placements or metrics. A comparison with available cut-points of older adult populations (REF Santos-Lozano) and other published cut-points of adult populations [13, 21, 34, 35] shows comparable values and, if applied to our data, a comparable performance, even if the cut-points were not generated specifically for an older population (see S6 Appendix). In our study, the mean age of the participants was 62.9 years, and while the population shows higher empirical MET values during the treadmill tests in comparison to the normative values in the PA compendium [31], cut-points derived from the general adult population might still be applicable in this age group.

In our study, we found differences in the results for VM counts and ENMO. The finding is substantiated by the work of Migueles and colleagues, who also found that activity counts were not comparable to raw accelerations [36] underlining the importance to report both kinds of metrics to allow for comparison of research results. Beyond ENMO and the use of cut-points, alternative metrics for raw acceleration have been proposed [37]. However, research is still ongoing and no ready-to-use solution is currently available [38, 39].

### Accelerometer placement

It showed that performance of different cut-points differed by investigated activity and placement. The intensity of the cycling activity, which predominantly involves movement in the lower body, was best estimated by the ankle placement. Likewise, the cleaning activity was best estimated by the wrist placement as the activity involved mostly movement of the upper body. The activity involving walking while carrying a bag of groceries was underestimated by all accelerometer placements. Overall, hip placement performed worst in classifying intensities of the free-living activities.

The agreement of non-dominant and dominant limbs as well as of correct vs. erroneous hip placements was high. The lack of difference between non-dominant and dominant wrist is well supported by previous research [40, 41], although some studies found statistically significant differences between both sides [36]. A recent study in older adults found incorrect accelerometer placements in more than 15% of the participants for one or more days during a seven day measurement period [42], emphasizing the importance of including erroneous placements in validation studies.

For activities where movement is not proportional to energy expenditure, accelerometers tend to misclassify the intensity [6, 43]. This is the likely reason for the underestimation by all placements of the load-bearing shopping activity, and the overestimation of the aerobic activity by wrist placement that involved a lot of arm movement. For the latter scenario, a combination of accelerometer placements could offer a solution, however, this places additional burden on the study subjects and might hamper compliance with the measurement. For wrist placement, which has become more and more popular due to a considerably higher compliance of study participants [23], it has to be noted that our data revealed a higher inter-individual variability of arm movement (wrist placement) compared to core body (hip placement) during activities (see also SD values in S4 Appendix).

### Strengths and limitations

The participants in the current study were healthy and without functional limitations, therefore, findings may not be generalizable to individuals with cardiovascular or metabolic conditions [44] and individuals with functional limitations [45]. Metabolic cost of ambulation increases with age, therefore, the standard MET calculation may underestimate PA intensity in older adults [44]. Evenson and colleagues suggest using individualized cut-points for older adult cohorts [46], which is likely unfeasible in larger studies [47]. Accelerometer cut-points for the vigorous intensity had to be linearly extrapolated mainly from data in lower intensities. Including faster walking speeds in the treadmill protocol was not deemed feasible for this older population. This puts an implicit assumption on the upper cut-off (vigorous PA) that the association between energy expenditure and accelerometer is linear also in the upper range. This might result in a misclassification of the accelerometer data. As only 5% of the activities were in the vigorous activity range, the extent of error is limited.

A particular strength of the study is the high variety of placements as well as the inclusion of VM counts and ENMO in one single study, which allows direct comparison of the resulting data. Further, the performance of the cut-points was tested using activities found to be typical for the age group 65–75 years within the OUTDOOR ACTIVE pilot study [48]. Cycling is very prevalent in Northern Germany for transport as well as for leisure [49]. Thus, the inclusion of cycling in the range of test activities is important to assess cut-points that are meant to be employed for measurement of free-living PA.

### Conclusions

The ability to assess PA intensity levels in older adults with the use of accelerometer cut-points allows the formulation of evidence-based PA recommendations. In this paper, we provided cut-points for older adults; however, the validity of assessing intensity levels by accelerometer measurements depends on placement, activity and investigated parameter. More research and innovative approaches are needed to obtain valid PA measurements across populations and age groups. There is a definite need for an objective assessment method for PA intensity which has high internal and external validity to minimize information bias while being acceptable to the study participants of large-scale epidemiological studies to prevent selection bias due to lack of compliance.

## Supporting information

S1 Appendix De-anonymized minimal data set of the study. (SAV)

S2 Appendix Protocol for the generation and validation of ActiGraph GT3X+ accelerometer cut-points for assessing physical activity intensity in older adults. (DOCX)

S3 Appendix Missing values of staged activities and treadmill test. (DOCX)

S4 Appendix Means and standard deviations of accelerometer VM counts and ENMO values at different walking speeds on the treadmill. (DOCX)

S5 Appendix Pearson correlations between vector magnitude (VM) counts and Euclidian norm minus one (ENMO) for different accelerometer placements. (DOCX)

S6 Appendix Comparison of OUTDOOR ACTIVE cut-points with published cut-points. (DOCX)
