# Supplementary material for: Generation and validation of ActiGraph GT3X+ accelerometer cut-points for assessing physical activity intensity in older adults. The OUTDOOR ACTIVE validation study
Source: PLoS One. 2021 Jun 3;16(6):e0252615. doi: 10.1371/journal.pone.0252615 (PMC8174693; doi:10.1371/journal.pone.0252615)
Supplement: S2 Appendix — (DOCX) [file pone.0252615.s002.docx]

**S2 Appendix: Protocol for the generation and validation of ActiGraph GT3X+ accelerometer cut-points for assessing physical activity intensity in older adults.**

**Before participant arrives**

- Calibrate breath-by-breath indirect calorimetry machine as per manufacturer’s guidelines
- Initialise accelerometers as per manufacturer’s guidelines
- Organise equipment

**Before testing**

- Welcome participant
- Bring them to the lab
- Explain protocol
- Complete consent form and medical questionnaire
- Discuss what activities can be done
- Take participants height and weight without shoes
- Bring participant and sit on the bed
- Moisten HR strap with water and attach to participant ensuring privacy and that participant is comfortable
- Put on the accelerometers: dominant + non-dominant wrist, hip, hip error (somewhere over the hip), dominant + non-dominant ankle (devise facing outside)
- Take BP while participant is sitting on bed - if high, take again after ‘reading’ activity
- Add participant details and ID to indirect calorimetry program
- Put breath-by-breath face mask with mouth piece on participant
- Attach sample line
- Begin testing

**Testing**

**Begin Reading protocol (3 mins)**

- Explain to participant the actions on the bed (reading magazine)
- Ask participant to sit comfortably on bed
- Maybe talk to them throughout the activity
- Take time and HR at beginning and end of activity

**Begin Treadmill protocol (20 mins)**

- Explain activity
- Ensure participant is happy to continue
- Help participant onto treadmill
- Show them the emergency stop button and attach the emergency cord to clothing
- Explain what the next steps are and ensure participant is happy to continue
- Fix sample line under head strap
- Increase treadmill incline to 1%
- Record time and HR
- Count to 3 and start treadmill very slowly
- Start increasing speed up to 3.5 km·h^-1^ gradually while ensuring participant is happy to continue
- Participant walks for 4 minutes at 3.5 km·h^-1^
- Increase speed every 4 minutes by 0.5 km·h^-1^ and record Time and HR
- After completing activity record final Time and HR
- Reduce treadmill speed and come to stop while ensuring participant is okay
- Ask if participant wants a break, water and to remove mask for 5-10 minutes

**Begin Shopping protocol (4 mins, 2-4kg)**

- Explain/demonstrate what the next activity is
- Test how much weight they need in the shopping bag
- Ask if participant is happy to begin shopping protocol
- *If participant had a break, reattach mask*
- *Put mask on participant and tighten, check for gaps*
- *Attach sample line*
- *Help participant onto treadmill*
- *Show them the emergency stop button*
- Check participant is ready to begin
- Hand participant shopping bag with weight (2-4kg)
- Increase treadmill incline to 1%
- Record time and HR
- Start increasing speed very carefully up to 2.5 km·h^-1^ gradually while ensuring participant is happy to continue, ask if you should increase the speed for normal walking (could be very different between participants)
- Participant walks for 4 minutes at their chosen speed
- After completing activity record final Time and HR
- Reduce treadmill speed and come to stop while ensuring participant is okay
- Ask if participant wants a break, water and to remove mask for some minutes

**Begin Cleaning protocol (4 minutes)**

- Explain/demonstrate what the next activity is
- Ask if participant is happy to begin brushing protocol
- *If participant had a break, reattach mask*
- *Put mask on participant and tighten, check for gaps*
- *Attach sample line*
- Check participant is ready to begin
- Hand participant the broom
- Record time and HR
- Encourage participant throughout the 4 minutes of brushing
- After completing activity record final Time and HR

**Begin Cycling protocol (4 minutes at chosen speed, resistance 2)**

- Explain/demonstrate what the next activity is
- Ask if participant is happy to begin cycling protocol
- *If participant had a break, reattach mask*
- *Put mask on participant and tighten, check for gaps*
- *Attach sample line - raised edge facing away from face*
- Help participant on to bike ensuring height of the saddle and handlebars are optimal
- Record time and HR
- Encourage participant throughout the 4 minutes of cycling
- After completing activity record final Time and HR and average power
- Ask if participant wants a break, water and to remove mask for some minutes

**Begin Aerobics protocol (4 mins)**

- Explain what the next activity is
- Ask if participant is happy to begin aerobics protocol
- *If participant had a break, reattach mask*
- *Take mouthpiece out of syringe and insert into mask*
- *Put mask on participant and tighten, check for gaps*
- *Attach sample line*
- Record Time and HR
- Show the activities:
  - 30sec. Walking in place
  - 30sec. Walking with shaking hands
  - 30 sec. Sidestep
  - 30 sec. Sidestep with arms on side
  - 30 sec. Rise knees
  - 30 sec. Rise knees with elbows to knees (right elbow to left knee)
  - 30 sec. Leg curl
  - 30 sec. Leg curl with arm bicep curl
- Encourage participant throughout
- Record time and HR at the end of aerobics protocol

**Ending Testing**

- Remove sample line, face mask, HR strap and accelerometers
- Give some water, if participant wants
- Thank them and ask if they have any questions
- Say goodbye
