# Supplementary material for: Generation and validation of ActiGraph GT3X+ accelerometer cut-points for assessing physical activity intensity in older adults. The OUTDOOR ACTIVE validation study
Source: PLoS One. 2021 Jun 3;16(6):e0252615. doi: 10.1371/journal.pone.0252615 (PMC8174693; doi:10.1371/journal.pone.0252615)
Supplement: S3 Appendix — (DOCX) [file pone.0252615.s003.docx]

**Appendix S3: Missing values of staged activities and treadmill test.**

| **Activities** | **Missing values (n)** | **Subject ID** |
| --- | --- | --- |
|  |  |  |
| **Staged activities** |  |  |
| Shopping | 1 | #6 |
|  |  |  |
| **Treadmill test** |  |  |
| 3.0 km·h^-1^ | 1 | #6 |
|  |  |  |
| 3.5 km·h^-1^ | 1 | #6 |
|  |  |  |
| 4.0 km·h^-1^ | 1 | #6 |
|  |  |  |
| 4.5 km·h^-1^ | 1 | #14 |
|  |  |  |
| 5.0 km·h^-1^ | 1 | #14 |
|  |  |  |

#6 Failure of VO_2_ measurement during treadmill activities; did not perform shopping activities;

#14 Did not perform two of the treadmill activities
