# Supplementary material for: Generation and validation of ActiGraph GT3X+ accelerometer cut-points for assessing physical activity intensity in older adults. The OUTDOOR ACTIVE validation study
Source: PLoS One. 2021 Jun 3;16(6):e0252615. doi: 10.1371/journal.pone.0252615 (PMC8174693; doi:10.1371/journal.pone.0252615)
Supplement: S4 Appendix — (DOCX) [file pone.0252615.s004.docx]

**S4 Appendix: Means and standard deviations of accelerometer VM counts and ENMO values at different walking speeds on the treadmill.**

| **Placement** | **Sex** | **Means (standard deviations) accelerometer VM counts at:** | | | | |
| --- | --- | --- | --- | --- | --- | --- |
|  |  | **3.0 km·h^-1^** | **3.5 km·h^-1^** | **4.0 km·h^-1^** | **4.5 km·h^-1^** | **5.0 km·h^-1^** |
|  |  |  |  |  |  |  |
| **Ankle dominant** | **Male** | 6678 (1011) | 7656 (1155) | 8752 (1269) | 10394 (1368) | 11700 (1450) |
|  | **Female** | 8216 (2068) | 8930 (1580) | 10351 (1602) | 11863 (1616) | 13115 (1187) |
|  |  |  |  |  |  |  |
| **Ankle non-dominant** | **Male** | 6345 (622) | 7558 (990) | 8758 (966) | 10336 (1186) | 11492 (1082) |
|  | **Female** | 8184 (2009) | 9078 (1446) | 10442 (1350) | 12003 (1330) | 13160 (876) |
|  |  |  |  |  |  |  |
| **Wrist dominant** | **All** | 3236 (2232) | 3482 (1618) | 4404 (1694) | 5004 (1861) | 5436 (1926) |
|  |  |  |  |  |  |  |
| **Wrist non-dominant** | **All** | 2624 (2043) | 2983 (1697) | 3630 (1663) | 4313 (1390) | 4695 (1506) |
|  |  |  |  |  |  |  |
| **Hip correct** | **All** | 1951 (629) | 2514 (611) | 3152 (649) | 3649 (488) | 4398 (557) |
|  |  |  |  |  |  |  |
| **Hip erroneous** | **All** | 1709 (592) | 2457 (618) | 3186 (595) | 3768 (448) | 4570 (536) |
|  |  |  |  |  |  |  |
| **Placement** | **Sex** | **Means (standard deviations) accelerometer ENMO values at:** | | | | |
|  |  | **3.0 km·h^-1^** | **3.5 km·h^-1^** | **4.0 km·h^-1^** | **4.5 km·h^-1^** | **5.0 km·h^-1^** |
|  |  |  |  |  |  |  |
| **Ankle dominant** | **Male** | 218 (48) | 271 (53) | 332 (59) | 399 (63) | 450 (62) |
|  | **Female** | 296 (74) | 345 (68) | 409 (69) | 467 (74) | 532 (64) |
|  |  |  |  |  |  |  |
| **Ankle non-dominant** | **Male** | 209 (50) | 262 (57) | 320 (51) | 389 (56) | 447 (67) |
|  | **Female** | 284 (80) | 330 (64) | 395 (67) | 453 (68) | 513 (70) |
|  |  |  |  |  |  |  |
| **Wrist dominant** | **All** | 95 (56) | 105 (53) | 128 (56) | 152 (69) | 171 (70) |
|  |  |  |  |  |  |  |
| **Wrist non-dominant** | **All** | 67 (49) | 86 (54) | 107 (63) | 128 (61) | 146 (67) |
|  |  |  |  |  |  |  |
| **Hip correct** | **All** | 55 (13) | 70 (12) | 87 (15) | 107 (17) | 129 (20) |
|  |  |  |  |  |  |  |
| **Hip erroneous** | **All** | 68 (18) | 82 (17) | 100 (18) | 120 (17) | 145 (20) |
|  |  |  |  |  |  |  |

VM vector magnitude

ENMO Euclidian norm minus one
