# Supplementary material for: Generation and validation of ActiGraph GT3X+ accelerometer cut-points for assessing physical activity intensity in older adults. The OUTDOOR ACTIVE validation study
Source: PLoS One. 2021 Jun 3;16(6):e0252615. doi: 10.1371/journal.pone.0252615 (PMC8174693; doi:10.1371/journal.pone.0252615)
Supplement: S5 Appendix — (DOCX) [file pone.0252615.s005.docx]

**S5 Appendix: Pearson correlations between vector magnitude (VM) counts and Euclidian norm minus one (ENMO) for different accelerometer placements.**

|  |  |  | **VM counts** | | | | | |  | **ENMO values** | | | | | |
| --- | --- | --- | --- | --- | --- | --- | --- | --- | --- | --- | --- | --- | --- | --- | --- |
|  |  |  | **Accelerometer placement** | | | | | |  | **Accelerometer placement** | | | | | |
|  |  |  | **Ankle dominant** | **Ankle non-dominant** | **Wrist dominant** | **Wrist non-dominant** | **Hip**  **correct** | **Hip erroneous** |  | **Ankle dominant** | **Ankle non-dominant** | **Wrist dominant** | **Wrist non-dominant** | **Hip**  **correct** | **Hip erroneous** |
| **VM counts** | **Accelerometer placement** | **Ankle dominant** |  | 0,996* | -0,077 | -0,017 | 0,333* | 0,322* |  | 0,871* | 0,870* | -0,070 | 0,004 | 0,379* | 0,389* |
|  |  | **Ankle non-dominant** |  |  | -0,072 | -0,010 | 0,343* | 0,325* |  | 0,866* | 0,870* | 0,478* | 0,557* | 0,389* | 0,743* |
|  |  | **Wrist dominant** |  |  |  | 0,952* | 0,541* | 0,558* |  | -0,047 | -0,068 | 0,953* | 0,908* | 0,321* | 0,337* |
|  |  | **Wrist non-dominant** |  |  |  |  | 0,589* | 0,609* |  | 0,031 | 0,013 | 0,908* | 0,950* | 0,382* | 0,387* |
|  |  | **Hip**  **correct** |  |  |  |  |  | 0,902* |  | 0,470* | 0,463* | 0,478* | 0,557* | 0,800* | 0,743* |
|  |  | **Hip erroneous** |  |  |  |  |  |  |  | 0,526* | 0,526* | 0,908* | 0,950* | 0,855* | 0,790* |
|  |  |  |  |  |  |  |  |  |  |  |  |  |  |  |  |
| **ENMO values** | **Accelerometer placement** | **Ankle dominant** |  |  |  |  |  |  |  |  | 0,990* | -0,011 | 0,083 | 0,635* | 0,601* |
|  |  | **Ankle non-dominant** |  |  |  |  |  |  |  |  |  | -0,039 | 0,059 | 0,635* | 0,581* |
|  |  | **Wrist dominant** |  |  |  |  |  |  |  |  |  |  | 0,925* | 0,318* | 0,363* |
|  |  | **Wrist non-dominant** |  |  |  |  |  |  |  |  |  |  |  | 0,385* | 0,408* |
|  |  | **Hip**  **correct** |  |  |  |  |  |  |  |  |  |  |  |  | 0,873* |
|  |  | **Hip erroneous** |  |  |  |  |  |  |  |  |  |  |  |  |  |
| * p<0.01 | | | | | | | | | | | | | | | |
