# Supplementary material for: Generation and validation of ActiGraph GT3X+ accelerometer cut-points for assessing physical activity intensity in older adults. The OUTDOOR ACTIVE validation study
Source: PLoS One. 2021 Jun 3;16(6):e0252615. doi: 10.1371/journal.pone.0252615 (PMC8174693; doi:10.1371/journal.pone.0252615)
Supplement: S6 Appendix — (DOCX) [file pone.0252615.s006.docx]

**Appendix S6: Comparison of OUTDOOR ACTIVE cut-points with published^a^ cut-points.**

**Proportion of physical activity intensities (sedentary/light-moderate-vigorous) in percent and intraclass correlation (2-way-mixed) for classification by accelerometer with reference**

| **VM counts** |  |  |  |  |
| --- | --- | --- | --- | --- |
|  | **VM cut-point moderate PA intensity** | **VM cut-point vigorous PA intensity** | **Proportion of intensities** | **ICC** |
| **Reference V0^2^** |  |  | 37-58-5 | - |
|  |  |  |  |  |
| **Hip placement** |  |  |  |  |
| OA cut-point | 2857 | 6799 | 73-25-2 | 0.25 |
| Santos-Lozano 2013 | 2751 | 9359 | 74-26-0 | 0.22 |
| Sasaki 2011^b^ | 2690 | 6167 | 73-24-3 | 0.24 |
|  |  |  |  |  |
| **Non-dominant wrist** |  |  |  |  |
| OA cut-point | 3268 | 7890 | 48-25-27 | 0.30 |
| Rhudy 2020^bc^ | 4836 | 8453 | 57-16-27 | 0.32 |
| Montoye 2020^b^ | 3941 | 5613 | 51-13-36 | 0.23 |
|  |  |  |  |  |
| **ENMO** |  |  |  |  |
|  | **ENMO cut-point moderate PA intensity** | **ENMO cut-point vigorous PA intensity** | **Proportion of intensities** | **ICC** |
| **Reference V0^2^** |  |  | 37-58-5 | - |
|  |  |  |  |  |
| **Hip placement** |  |  |  |  |
| OA cut-point | 82 | 191 | 84-16-0 | 0.17 |
| Hildebrand 2014^b^ | 69.1 | 258.7 | 76-24-0 | 0.20 |
|  |  |  |  |  |
| **Non-dominant wrist** |  |  |  |  |
| OA cut-point | 100 | 245 | 56-24-20 | 0.40 |
| Hildebrand 2014^b^ | 100.6 | 428.8 | 57-36-7 | 0.33 |
|  |  |  |  |  |

^a^Cut-points by Barnett 2016 were only available for moderate intensity and were, therefore, not included in this table.

^b^Cut-points were derived from an adult population and are not specific for older adults.

^c^Cut-points were assessed for left wrist irrespective of dominance.

ENMO Euclidian norm minus one

ICC Intraclass correlation

OA Outdoor active

VM Vector magnitude
